# Phytochemical Profiling of the Halophyte *Suaeda vera* and Antileishmanial Evaluation of Its Extracts and Selected Metabolites, with In Silico ADMET Analysis

**DOI:** 10.3390/molecules31213967

**Published:** 2026-11-03

**Authors:** Elea Serrano-Martínez, Estela Melcón-Fernández, Cathy Soh-Kamdjo, María-Cristina González-Montero, Julia Andrés-Rodríguez, Linda Majdoub-Mathlouthi, Mariem Rouatbi, Mouna Friha, Evangelia Sossidou, Yolanda Pérez-Pertejo, Rosa M. Reguera, Carlos García-Estrada, Rafael Balaña-Fouce

**Affiliations:** 1 Departamento de Ciencias Biomédicas, Facultad de Veterinaria, Universidad de León, Campus de Vegazana s/n, 24007 León, Spain; eserm@unileon.es (E.S.-M.); emelf@unileon.es (E.M.-F.); csohk@unileon.es (C.S.-K.); magom@unileon.es (M.-C.G.-M.); jandr@unileon.es (J.A.-R.); myperp@unileon.es (Y.P.-P.); rmregt@unileon.es (R.M.R.); 2 LR Gestion des Ressources Animales et Environnementales dans le Semi-Aride, Institut Supérieur Agronomique de Chott Mariem, Université de Sousse, Sousse 4042, Tunisia; yalinmaj@gmail.com (L.M.-M.); rouatbi.myriam@yahoo.fr (M.R.); friha.mouna@gmail.com (M.F.); 3 Veterinary Research Institute, Hellenic Agricultural Organization, DIMITRA, 57001 Thessaloniki, Greece; sossidou@elgo.gr; 4 Instituto de Biomedicina (IBIOMED), Universidad de León, Campus de Vegazana s/n, 24007 León, Spain; 5 IBIOLEÓN (Instituto de Investigación Biosanitaria de León), Hospital de León, Altos de Nava s/n, 24071 León, Spain

**Keywords:** halophytes, *Leishmania*, organoids, amastigotes, alkaloids, secondary metabolites, plant natural products

## Abstract

Plant-derived secondary metabolites are a promising source of new anti-infective agents owing to their remarkable chemical diversity and broad spectrum of biological activities. Among them, halophytes are particularly promising because adaptation to saline environments promotes the accumulation of structurally diverse secondary metabolites. In the present study, organic extracts from four Tunisian halophytic species collected across different seasons were evaluated for their activity against *Leishmania infantum* axenic amastigotes, the etiological agent of visceral leishmaniasis in the Mediterranean Basin. Among the species tested, *Suaeda vera* was the only one to exhibit significant antileishmanial activity. Mass spectrometry-based phytochemical profiling of the bioactive extracts resulted in the putative annotation of 53 secondary metabolites and revealed qualitative differences in their annotated chemical composition among the seasonal samples. Three compounds putatively annotated in the active extracts—demethylzeylasteral, caffeic acid, and protocatechuic aldehyde—were subsequently evaluated individually using commercially available reference compounds and exhibited EC_50_ values of 0.20, 1.09, and 14.56 μM, respectively. Caffeic acid displayed the most favorable overall profile, combining potent antileishmanial activity with a high selectivity index (SI > 91.7) in mouse intestinal organoids. In vitro assays revealed that none of the three compounds inhibited trypanothione reductase-associated activity. Furthermore, in silico drug-likeness and ADMET analyses suggested that caffeic acid may have a comparatively favorable predicted pharmacokinetic and toxicity profile among the three compounds evaluated. Collectively, these findings highlight *S. vera* as a promising source of antileishmanial metabolites and establish caffeic acid as the most promising hit compound for further investigation.

## 1. Introduction

Plants represent one of the richest sources of structurally diverse bioactive molecules owing to their remarkable metabolic diversity and their ability to synthesize a wide range of secondary metabolites [1]. These compounds play key roles in plant defense and adaptation to environmental stress and exhibit a broad range of biological activities, including antioxidant, antimicrobial, anti-inflammatory, and anticarcinogenic effects [2]. Consequently, plant-derived bioactive compounds have attracted considerable interest in the food, pharmaceutical, and cosmetic industries, as well as in the development of functional ingredients and natural products with potential health benefits [3]. Despite the remarkable success of natural products in drug discovery, most plant metabolites remain unexplored, highlighting the enormous potential of plants as a reservoir of novel bioactive scaffolds.

Among the immense diversity of plant species, halophytes have emerged as particularly attractive sources of bioactive natural products. In the southern Mediterranean region, halophytic plants have traditionally been used as forage resources in extensive livestock production systems, particularly for sheep, goats and camels [4,5,6]. These plants have evolved to survive in highly saline environments by developing complex physiological and biochemical adaptations, including the enhanced biosynthesis and accumulation of secondary metabolites that protect against oxidative, osmotic, and environmental stress [7]. Consequently, halophytes are often enriched in structurally diverse phenolic compounds, flavonoids, terpenoids, alkaloids, coumarins, tannins, saponins, and other specialized metabolites with documented antioxidant, antimicrobial, anti-inflammatory, and antiproliferative properties [8,9]. Furthermore, the production and relative abundance of these metabolites are strongly influenced by environmental conditions, including salinity, water availability, temperature, and solar radiation, resulting in seasonal variations in the phytochemical composition and biological activity of halophytic species [10,11]. These characteristics make halophytes a particularly attractive source of structurally diverse natural products for drug discovery and other pharmaceutical applications [12,13].

Natural products, including those derived from halophytic species, have been extensively investigated as sources of novel antimicrobial and antiparasitic agents [14,15]. Among the diseases that would particularly benefit from the discovery of new bioactive compounds is leishmaniasis, a group of neglected tropical diseases caused by protozoan parasites of the genus *Leishmania* and transmitted to humans through the bite of infected phlebotomine sandflies [16]. More than 20 *Leishmania* species are pathogenic to humans. *Leishmania donovani* and *Leishmania infantum* are the principal causative agents of visceral leishmaniasis, the most severe clinical form of the disease, whereas species such as *L. major*, *L. tropica*, *L. mexicana*, and *L. braziliensis* are mainly responsible for cutaneous and mucocutaneous leishmaniasis [17,18]. According to the World Health Organization (WHO), between 50,000 and 90,000 new cases of visceral leishmaniasis occur annually, although only 25–45% are officially reported. Without treatment, the disease is fatal in more than 95% of affected patients [16].

The current treatment of leishmaniasis relies on a limited number of outdated drugs, including pentavalent antimonials, amphotericin B, miltefosine, and paromomycin. However, their clinical use is constrained by important drawbacks such as toxicity, prolonged treatment regimens, high cost, parenteral administration, variable efficacy depending on the infecting species and geographical region, and the emergence of drug-resistant parasites [19,20,21]. Moreover, climate change and increasing human mobility are expected to facilitate the expansion of phlebotomine sandfly vectors into previously non-endemic regions, potentially increasing the global burden of leishmaniasis in the coming decades [18]. Consequently, the identification of new, safe, and effective antileishmanial compounds remains a high priority [21].

Among the molecular targets currently explored for antileishmanial drug discovery, trypanothione reductase (TryR) has attracted considerable attention because it plays a central role in maintaining the unique trypanothione-dependent redox metabolism of *Leishmania*. This enzyme, which is absent in mammalian cells, is essential for protecting the parasite against oxidative stress by reducing oxidized trypanothione (TS_2_) to its active dithiol state in the presence of NADPH, making it an attractive target for the development of selective antileishmanial agents [22,23,24]. Nevertheless, despite the identification of numerous natural products with antileishmanial activity, relatively few studies have attempted to identify their molecular targets, limiting the translation of these compounds into drug discovery programs.

Based on these considerations, the present study aimed to evaluate the antileishmanial activity of extracts obtained from halophytic plants collected from Kerkennah Islands (eastern coast of Tunisia). The chemical composition of the bioactive extracts was characterized by mass spectrometry, resulting in the putative annotation of secondary metabolites. Selected compounds were subsequently evaluated for their antileishmanial activity and cytotoxicity in a 3D (three-dimensional) mouse intestinal organoid model, and their potential effects on TryR-associated activity were explored in a preliminary assay. Predicted drug-likeness, pharmacokinetic, and toxicity profiles were also assessed to identify promising candidates for further antileishmanial drug development.

## 2. Results

### 2.1. Antileishmanial Activity of Plant Extracts

Halophytes, typically utilized for local extensive small ruminant grazing, were harvested from the Kerkennah Islands (Tunisia) and botanically identified before processing. A total of nine samples (TN1–TN9) were obtained by drying and milling plant material from four species collected across multiple seasons (Table 1).

**Figure 1:**
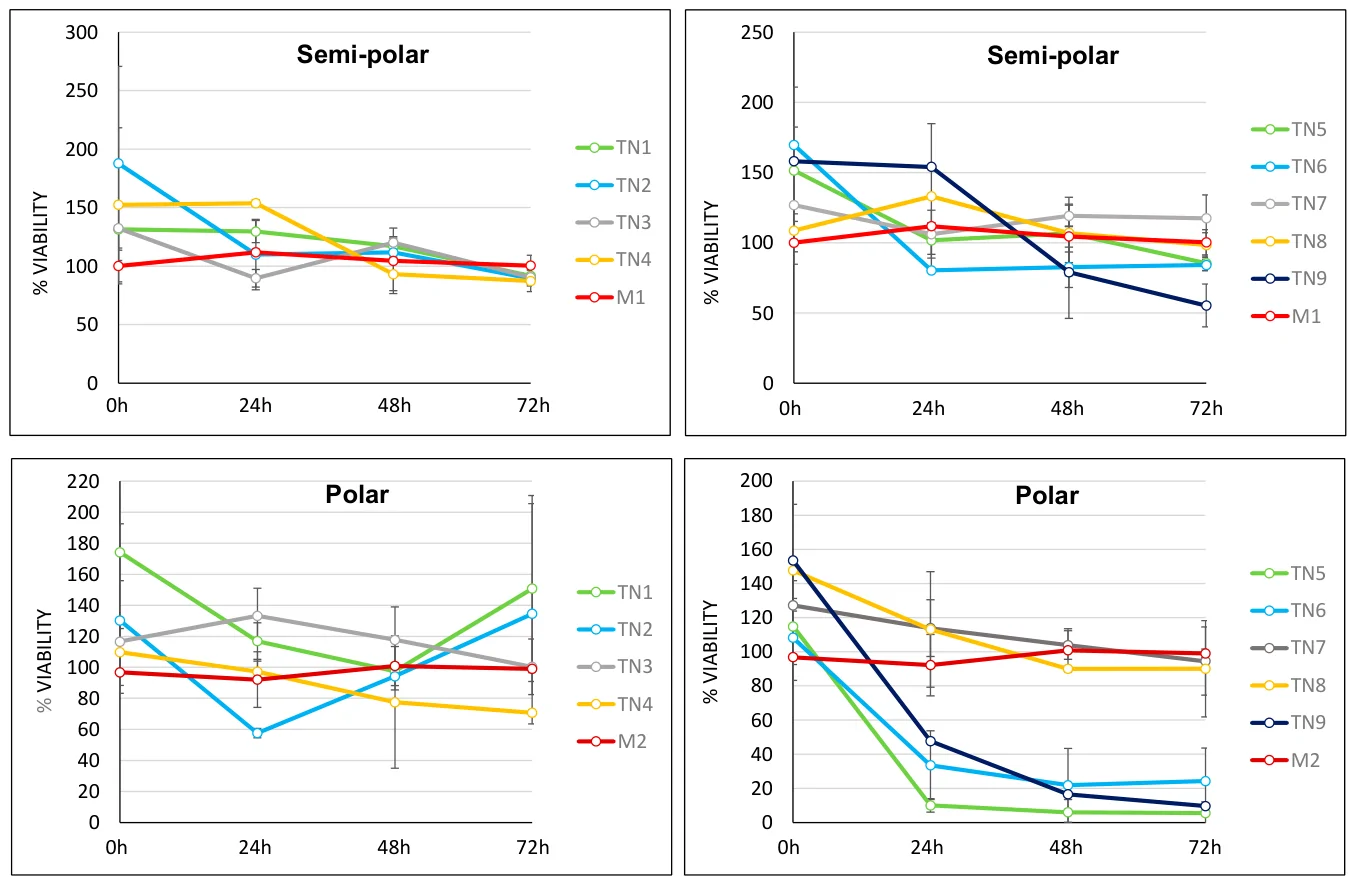
Efficacy of semi-polar (**upper**) panels and polar (**lower**) panels extracts from samples TN1–TN9 against *L. infantum*-iRFP axenic amastigotes. M1 (methyl tert-butyl ether:methanol 3:1, *v*/*v*) and M2 (water:methanol 3:1, *v*/*v*) correspond to the extraction solvent controls. Parasite viability (%) was measured at 0, 24, 48, and 72 h. Results show the mean ± SD of three independent experiments with three technical replicates each.

Polar and semi-polar extracts were prepared from TN1-TN9 samples as indicated in Section 4. Activity against *L. infantum*-iRFP axenic amastigotes, expressing infrared fluorescent protein (iRFP), was assessed for up to 72 h by means of reduction in fluorescence emitted by the parasites, which was used as a proxy for parasite viability (Figure 1).

The semi-polar extracts exhibited lower antiparasitic activity than the polar extracts. Among the semi-polar extracts, only those obtained from *S. vera* collected in winter (TN9) achieved a significant reduction in parasite viability, reaching 55.30% after 72 h of incubation. The polar extracts of *L. guyonianum* (TN1, TN2), *S. europaea* (TN3, TN4), and *H. portulacoides* (TN7, TN8) generally exhibited weak or no antiparasitic activity, with viability values remaining above 75% at most of the evaluated time points. Although sample TN2 (*L. guyonianum*, spring) produced a marked decrease in viability at 24 h, this effect was not sustained over time, reverting at 48 and 72 h to values even higher than those observed for the M2 (water:methanol 3:1, *v*/*v*) solvent control. Likewise, sample TN4 (*S. fruticosa*, spring) exerted a mild antileishmanial effect, reducing viability to 70.73% after 72 h. In contrast, the polar extracts of *S. vera* (TN5, TN6, and TN9) exhibited the highest antiparasitic activity among all the extracts tested. Sample TN5 (summer) reduced amastigote viability to 9.98% at 24 h, 5.97% at 48 h, and 5.53% at 72 h. Samples TN6 (spring) and TN9 (winter) also decreased parasite viability to 24.20% and 9.62%, respectively, after 72 h of incubation (Figure 1).

### 2.2. Cytotoxicity of Antileishmanial Plant Extracts in Intestinal Organoids

**Figure 2:**
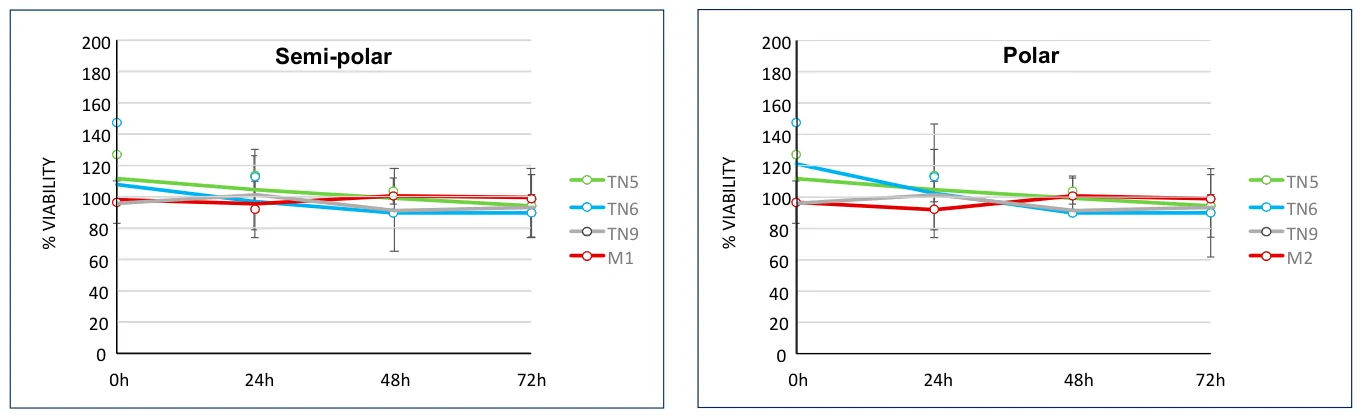
Cytotoxicity of the semi-polar and polar extracts of *S. vera* (TN5, TN6, and TN9 samples). Solvent controls M1 (methyl tert-butyl ether:methanol 3:1, *v*/*v*) and M2 (water:methanol 3:1, *v*/*v*) are shown in red. Cell viability (%) was assessed at 0, 24, 48, and 72 h. Results show the mean ± SD of three independent experiments with three technical replicates each.

The safety of the plant extracts that showed efficacy against *L. infantum*-iRFP axenic amastigotes (*S. vera* TN5, TN6, and TN9) was evaluated for up to 72 h in murine intestinal organoids obtained as described in Section 4. Cell viability was determined using the alamarBlue assay. As shown in Figure 2, none of the extracts tested caused a significant decrease in cell viability, indicating that they are not cytotoxic in this 3D culture model.

### 2.3. Mass Spectrometric Characterization of Bioactive and Non-Toxic S. vera Extracts

Mass spectrometry-based phytochemical profiling of *S. vera* extracts TN5, TN6, and TN9 revealed differences in their chemical composition depending on both the polarity of the extraction solvent and the sampling time. Candidate metabolites detected in positive and negative ionization modes with a similarity score above 70% to compounds in the reference databases were selected for further analysis. This relatively permissive threshold was adopted due to the exploratory nature of the study and the limited representation of halophyte metabolites in available databases. After discarding primary metabolites, the putatively annotated secondary metabolites present in the polar and semi-polar extracts are listed in Supplementary Tables S1 and S2, respectively.

A total of 53 distinct secondary metabolites were putatively annotated in the extracts of *S. vera.* These annotations encompassed several chemical categories including flavonoids, terpenoids, alkaloids, phenylpropanoids, phenolic compounds, coumarins, chromones, lipids, and natural peptides. Based on the putative annotations, polyphenols and terpenoids were the most represented chemical groups, mainly comprising flavonoids, triterpenoids, and triterpenoid saponins (Table 2).

Most compounds were detected in both polar and semi-polar extracts, although some metabolites were found exclusively in a specific extract type. For example, asiatic acid and thymol were only detected in the polar and semi-polar extracts, respectively (Supplementary Tables S1 and S2).

In the polar extracts (Supplementary Table S1), three compounds were consistently putatively annotated in all samples: protocatechuic aldehyde, a phenolic aromatic aldehyde, and two flavonoids, isorhamnetin, and narcissoside. Similarly, protocatechuic aldehyde was putatively annotated in all semi-polar extracts (Supplementary Table S2), suggesting that this phenolic aromatic aldehyde was consistently annotated across the *S. vera* samples analyzed. The polar extracts of the spring and summer samples exhibited similar chemical profiles, with most of the annotated metabolites corresponding to phenolic acids and their derivatives or flavonoids. In contrast, the winter sample showed a greater number of putatively annotated metabolites, including not only flavonoids but also alkaloids, such as isorhynchophylline, and terpenoids, including asiatic acid, betulonic acid, and ursolic acid (Supplementary Table S1). Likewise, the semi-polar extracts (Supplementary Table S2) displayed higher chemical diversity in the winter sample than in the spring and summer samples. Spring and summer extracts contained compounds that were absent from their corresponding polar extracts, such as demethylzeylasteral and nodakenin, whereas the winter extract included additional annotations, including peiminine and thymol.

Overall, the winter samples showed the highest numbers of putatively annotated metabolites (*n* = 24 and *n* = 22 in the polar and semi-polar extracts, respectively). In contrast, spring and summer extracts showed lower and comparable numbers of annotations, with 11 and 8 putatively annotated metabolites in the polar extracts and 13 in the semi-polar extracts for both seasons (Supplementary Tables S1 and S2).

### 2.4. In Vitro Efficacy and Safety of the Compounds Putatively Annotated in S. vera Extracts

**Figure 3:**
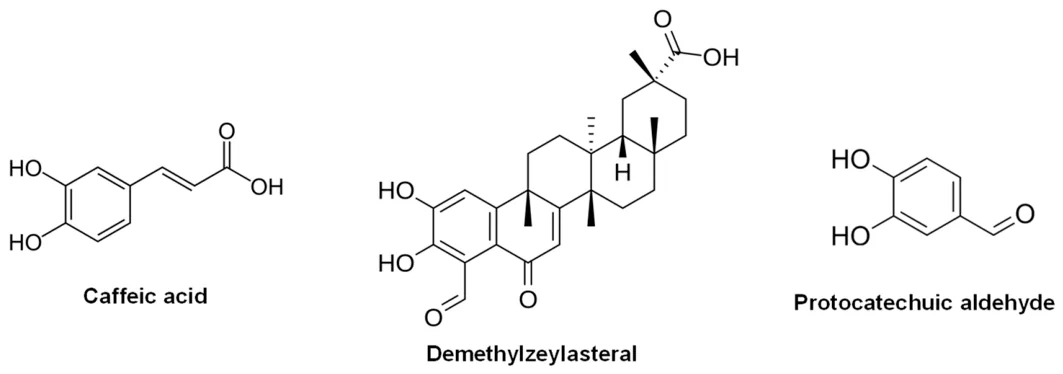
Chemical structure of caffeic acid, demethylzeylasteral, and protocatechuic aldehyde.

Following the putative annotation of candidate active compounds in the *S. vera* extracts, pure reference standards were purchased from MedChemExpress (Sollentuna, Sweden) and Merck (Darmstadt, Germany). Their leishmanicidal activity was subsequently evaluated against axenic amastigotes of *L. infantum*-iRFP in a single-concentration screen at 20 μM and 1 μM. In this initial screen, only three compounds exceeded the pre-established threshold of a >70% reduction in parasite viability at 20 μM: caffeic acid (phenolic compound), protocatechuic aldehyde (3,4-dihydroxybenzaldehyde) (phenolic compound), and demethylzeylasteral (terpene) (Figure 3). Caffeic acid and demethylzeylasteral also yielded similar results at 1 μM.

**Figure 4:**
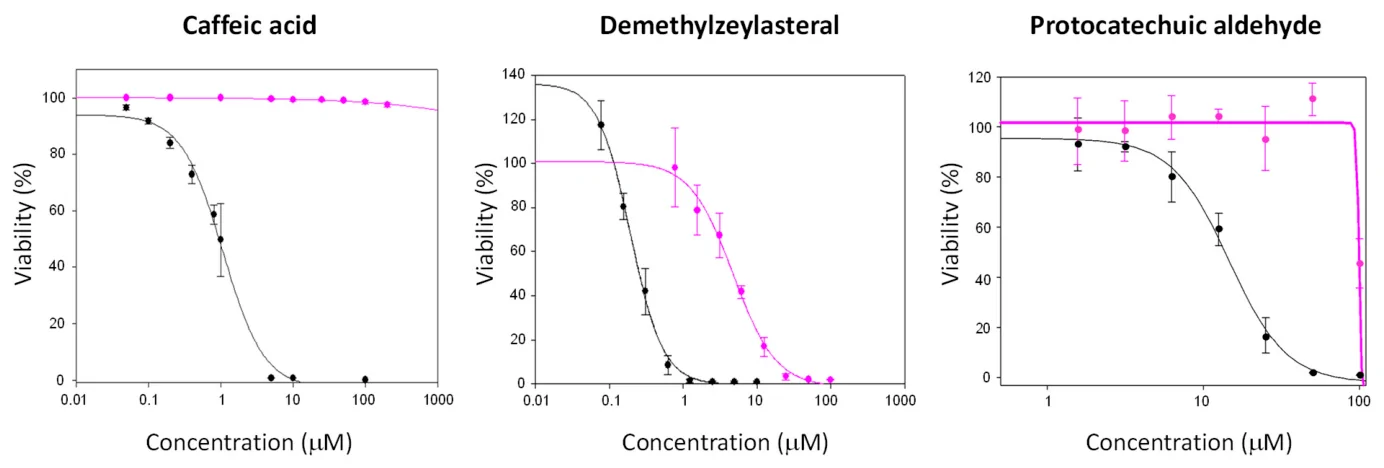
Dose–response curves for caffeic acid, demethylzeylasteral, and protocatechuic aldehyde against *L. infantum*-iRFP amastigotes (black lines) and murine intestinal organoids (pink lines). Percentage viability (*Y*-axis) was plotted against compound concentration (μM) (*X*-axis in logarithmic scale). The curves were generated using SigmaPlot software (version 10.0) based on data obtained after 72 h of incubation in the presence of each compound. Data are presented as the mean ± SD from three different experiments, each carried out in triplicate.

Dose–response curves were prepared for these three compounds using a range of increasing concentrations after testing their bioactivity against axenic amastigotes of *L. infantum*-iRFP (efficacy) and their cytotoxicity in murine intestinal organoids (safety) (Figure 4). Parasite cell viability was determined by measuring infrared fluorescence emission, while organoid viability was determined using the alamarBlue assay, as described in Section 4.

Based on the dose–response curves shown in Figure 4, EC_50_ (half maximal effective concentration) values for *L. infantum*-iRFP (efficacy) and CC_50_ values (half maximal cytotoxic concentration) for mouse intestinal organoids (safety) were determined and allowed for the calculation of the selectivity index (SI) using the CC_50_/EC_50_ ratio (Table 3).

Demethylzeylasteral was the most potent compound, followed by caffeic acid and protocatechuic aldehyde. However, potency and selectivity differed substantially among the compounds. Although demethylzeylasteral exhibited the lowest EC_50_, its lower CC_50_ (4.78 ± 0.68 μM) resulted in a SI of 23.9. In contrast, caffeic acid showed a CC_50_ > 100 μM and an SI > 91.74, indicating a more favorable selectivity profile. Protocatechuic aldehyde also showed a CC_50_ > 100 μM, but its lower potency resulted in an SI > 6.86. According to these data and considering the criteria proposed for visceral leishmaniasis (Hit: EC_50_ ≤ 10 μM and SI ≥ 10; Lead: EC_50_ ≤ 1 μM and SI ≥ 50) [25,26], caffeic acid and demethylzeylasteral were considered as hit compounds.

Since TryR is a validated molecular target involved in the maintenance of parasite redox homeostasis [24,27], as a preliminary assessment of a possible mechanism of action, caffeic acid, demethylzeylasteral, and protocatechuic aldehyde were evaluated for their effects on TryR-associated activity using *L. infantum*-iRFP protein extracts. None of the compounds inhibited the measured activity at the concentrations tested (25–100 μM; Supplementary Figure S1). However, as the assay was performed using protein extracts rather than purified recombinant TryR, these results do not rule out direct inhibition of the enzyme.

### 2.5. Predictive Drug-likeness and ADMET of the Selected Compounds

Predictive ADMET of caffeic acid, demethylzeylasteral, and protocatechuic aldehyde, was analyzed in silico using SwissADME and pkCSM (Table 4).

The ADMET properties of caffeic acid, demethylzeylasteral, and protocatechuic aldehyde were evaluated in silico using SwissADME and pkCSM (Table 4). All three compounds complied with Lipinski’s rule of five [28]. Caffeic acid and protocatechuic aldehyde were predicted to have high human intestinal absorption and not to be P-glycoprotein substrates, whereas demethylzeylasteral was predicted to have low intestinal absorption and to be a P-glycoprotein substrate. Regarding metabolism, caffeic acid was predicted to inhibit CYP1A2 and protocatechuic aldehyde to inhibit CYP3A4, while demethylzeylasteral was not predicted to inhibit any of the five CYP450 isoforms evaluated. A catechol_A PAINS alert was reported for all three compounds. None were predicted to be hepatotoxic, although protocatechuic aldehyde was predicted to be Ames-positive. The predicted acute oral toxicity values were of a similar order of magnitude for the three compounds (Table 4). These findings are computational predictions and require experimental validation; they should not be interpreted as confirmed pharmacokinetic or toxicological outcomes.

## 3. Discussion

The growing emergence of antimicrobial resistance is recognized by the WHO as one of the major public health threats of the coming decades, as it compromises the effectiveness of previously successful therapies and hinders the control of infectious diseases. In parallel, climate change is driving human population movements and facilitating the geographical expansion of vectors of neglected tropical diseases into regions where they are currently absent, which is expected to increase the global incidence of these diseases in the coming years. In this context, the identification of novel bioactive compounds with the potential to serve as therapeutic or prophylactic agents against diseases such as leishmaniasis has become an urgent priority [18].

Halophytes constitute a promising source of antimicrobials—including antileishmanial agents—as their adaptation to saline environments is frequently associated with the accumulation of a structurally diverse array of secondary metabolites absent in non-halophytic species. Nevertheless, the antileishmanial potential of this group of plants remains largely unexplored. In line with this hypothesis, extracts from the halophyte *Atriplex halimus*—a European fodder shrub of the Chenopodiaceae family—have previously shown modest activity against *Leishmania* [29].

In the present study, extracts from four halophytic species harvested in the Tunisian Kerkennah Islands were evaluated, and *S. vera* was the only species displaying significant antileishmanial activity. To the best of our knowledge, this is the first report describing the antileishmanial properties of this plant. Species of the genus *Suaeda*—Mediterranean halophytes belonging to the Amaranthaceae family that are adapted to saline soils and serve as a vital forage resource for small ruminants in North Africa—remain unexplored against *Leishmania*, despite reported antimicrobial activity in congeneric species such as *S. monoica*, *S. vermiculata*, and *S. pruinosa* [30,31,32]. Furthermore, *S. vera* has long been used in the traditional medicine of arid and semi-arid regions and is known to contain phenolic compounds, flavonoids, terpenoids, and saponins with antioxidant, anti-inflammatory, and antimicrobial properties [33]. The present study confirmed the antileishmanial activity of the polar extracts obtained from *S. vera* collected during spring (TN6), summer (TN5), and winter (TN9). Phytochemical profiling revealed differences in the qualitative composition of the putatively annotated metabolites among the *S. vera* samples collected in spring, summer, and winter, with the winter extract showing the highest number of annotations. Such seasonal fluctuations have previously been described in halophytic species and are thought to reflect adaptive responses to environmental stress that activate defense pathways and stimulate secondary metabolite biosynthesis [34,35]. Interestingly, despite the higher number of metabolites putatively annotated in the winter extract, the strongest antileishmanial activity was observed for the summer extract. This observation indicates that the number of metabolite annotations alone does not explain the differences in biological activity among the extracts. However, the contribution of individual metabolites to these differences and their concentrations remain to be established. Factors such as temperature, light availability, water availability, and salinity can influence the biosynthesis and accumulation of specialized metabolites, including phenolic compounds and flavonoids. In fact, the combined effects of intense solar radiation, water deficit, and increased soil salinity during summer are known to stimulate the biosynthesis and accumulation of secondary metabolites involved in stress adaptation [10,36], which may account for the enhanced biological activity observed in the summer extract. As these environmental variables were not directly measured in the present study, their contribution to the observed seasonal differences cannot be established. Since all three active polar extracts shared antileishmanial activity, one or more metabolites common to these extracts may contribute to the observed effect. However, the present qualitative profiling data do not allow us to determine whether differences in the concentrations of individual metabolites account for the differences in antileishmanial activity. Targeted quantitative metabolomic analyses will be required to test this hypothesis and identify the compounds primarily responsible for the biological activity. These observations are specific to the *S. vera* samples analyzed from spring, summer, and winter and should not be interpreted as establishing a general seasonal pattern for the species.

A total of 53 secondary metabolites were putatively annotated in the *S. vera* extracts. Among the three selected compounds evaluated individually, demethylzeylasteral exhibited the highest antileishmanial activity. Interestingly, although the semipolar spring extract (TN6), in which this compound was putatively annotated, showed only weak activity against *Leishmania*, the commercially obtained compound displayed a remarkably low EC_50_ value against axenic amastigotes. This discrepancy may reflect the complexity of the crude extract, including possible interactions with other metabolites that could attenuate or modify its biological activity. However, the contribution of these factors cannot be established from the present data. Conversely, synergistic interactions among compounds present in the more active extracts cannot be excluded, highlighting the complexity of phytochemical mixtures and the need for future bioactivity-guided fractionation studies. Demethylzeylasteral is a pentacyclic 6-oxophenolic triterpenoid previously reported to possess anti-inflammatory and antimicrobial activities, particularly against Gram-positive bacteria such as *Bacillus subtilis* [37,38,39]. To the best of our knowledge, this is the first study demonstrating its activity against *Leishmania*. Its antimicrobial activity has been associated with inhibition of N-acetyl-D-glucosamine incorporation, leading to disruption of bacterial cytoplasmic membrane integrity [37]. The antibacterial activity of demethylzeylasteral suggests that membrane-associated mechanisms may contribute to its biological activity. Although the plasma membrane of *Leishmania* differs substantially from that of bacteria, its unique lipid composition and abundance of surface glycoconjugates [40] also make it a potential target for membrane-active compounds [41]. Therefore, membrane perturbation cannot be excluded as a contributing mechanism underlying the antileishmanial activity of demethylzeylasteral. In addition to its direct leishmanicidal activity, demethylzeylasteral may exert beneficial host-directed effects. Previous studies have shown that this compound modulates signaling pathways such as NF-κB and TGF-β, both of which are manipulated by *Leishmania* to evade host immune responses [39]. Although the precise molecular targets of demethylzeylasteral remain to be elucidated, its combined antiparasitic and immunomodulatory properties make it an attractive scaffold for the development of new antileishmanial agents.

Caffeic acid, putatively annotated in the winter polar extract of *S. vera* (TN9), emerged as the most promising lead compound in this study. Although its antileishmanial potency was lower than that of demethylzeylasteral, its excellent SI value (>91.7), together with its favorable predicted pharmacokinetic profile and previous evidence of in vivo efficacy, make it a particularly attractive candidate for further development. It should be noted that the antileishmanial activity reported in this study was evaluated against axenic amastigotes. Further validation using intracellular amastigote models is therefore required to confirm the activity of caffeic acid under more physiologically relevant conditions and to better assess its therapeutic potential. Previous studies have identified *L. infantum* arginase as a molecular target of caffeic acid [42]. *L. infantum* arginase catalyzes the hydrolysis of L-arginine to L-ornithine, the first committed step in polyamine biosynthesis. This pathway is essential for parasite proliferation, differentiation, oxidative stress defense through trypanothione biosynthesis, and infectivity [43,44,45]. Caffeic acid inhibited approximately 57% of arginase activity at a concentration of 100 μM [42], while molecular docking analyses confirmed a stable interaction between the compound and the enzyme active site [46]. The antileishmanial activity of caffeic acid has been previously demonstrated against several *Leishmania* species [42,47], although with substantially higher EC_50_ values than those obtained in the present study. These differences are most likely explained by variations in the experimental models employed, including parasite species and developmental stage, as well as the use of intracellular versus axenic amastigotes. Moreover, the therapeutic potential of caffeic acid has already been supported by in vivo studies showing a significant reduction in lesion development in BALB/c mice infected with *L. amazonensis* [47]. Beyond its direct effects on parasite metabolism, caffeic acid has also been reported to enhance host antimicrobial responses. It increases nitric oxide production by activated macrophages, counteracting one of the immune evasion strategies employed by *Leishmania*; enhances macrophage iron sequestration, thereby restricting parasite access to this essential micronutrient; and promotes the generation of reactive oxygen species (ROS), contributing to oxidative stress-mediated parasite killing [48,49]. Collectively, these findings suggest that the antileishmanial activity of caffeic acid is likely multifactorial, involving both direct inhibition of parasite metabolic pathways and modulation of host immune defenses.

Finally, protocatechuic aldehyde exhibited moderate antileishmanial activity and relatively low SI. Although its activity against *Leishmania* has not previously been reported, its structural analog, protocatechuic acid, has been shown to display modest activity against *L. infantum* (IC_50_ = 32 μM) [50], suggesting that this class of phenolic compounds possesses intrinsic antileishmanial potential. Importantly, protocatechuic aldehyde was putatively annotated in all three *S. vera* extracts that exhibited antileishmanial activity and also demonstrated activity when tested as a purified compound. Together, these findings support the hypothesis that protocatechuic aldehyde contributes, at least in part, to the biological activity of the extracts. However, because plant extracts are chemically complex mixtures, the observed activity cannot be attributed exclusively to this metabolite, and synergistic or antagonistic interactions with other constituents are likely to influence the overall leishmanicidal effect.

To gain further insight into the mechanism underlying the antileishmanial activity of the three selected compounds, their ability to inhibit TryR was evaluated. TryR is one of the best-characterized molecular targets for antileishmanial drug discovery [24] because of its essential role in maintaining intracellular redox homeostasis. Given that caffeic acid has been reported to promote ROS generation and oxidative stress, inhibition of TryR was considered a plausible mechanism underlying its antileishmanial activity. However, none of the three compounds produced clear inhibition of TryR-associated activity in *L. infantum* protein extracts under the experimental conditions tested. However, because purified recombinant TryR was not used, these results do not conclusively establish whether the compounds directly inhibit the enzyme. Further biochemical studies with purified TryR will be required to clarify its potential involvement in their antileishmanial activity. Therefore, the oxidative stress associated with caffeic acid cannot be attributed to direct TryR inhibition on the basis of the present results. ROS accumulation may instead result from indirect effects on parasite metabolism or from modulation of host cell antimicrobial responses, including enhanced ROS, nitric oxide, and TNF-α production by infected macrophages, all of which have previously been reported for caffeic acid [42,49]. Similarly, although no clear inhibition of TryR-associated activity was observed for demethylzeylasteral or protocatechuic aldehyde, the molecular mechanisms underlying their antileishmanial activity remain to be elucidated.

The in silico evaluation provided additional insights into the potential developability and limitations of the three active compounds. All three complied with Lipinski’s rule of five, although this does not establish their suitability as oral drug candidates. Caffeic acid and protocatechuic aldehyde were predicted to have high human intestinal absorption and not to be P-glycoprotein substrates, whereas demethylzeylasteral was predicted to have low intestinal absorption and to be a P-glycoprotein substrate, suggesting that its absorption and exposure may require further investigation. The predicted inhibition of CYP1A2 by caffeic acid and CYP3A4 by protocatechuic aldehyde also warrants experimental assessment. In addition, protocatechuic aldehyde was predicted to be Ames-positive, highlighting a potential mutagenicity concern that requires further evaluation. Although none of the compounds was predicted to be hepatotoxic, these computational results do not establish their safety in vivo. SwissADME also identified a catechol_A PAINS alert for all three compounds. Such alerts indicate a potential risk of assay interference but do not, by themselves, demonstrate nonspecific activity or invalidate the observed antileishmanial effects. Nevertheless, orthogonal assays will be needed to assess the specificity of these effects, particularly for caffeic acid. Overall, the three compounds exhibited distinct predicted ADMET profiles and potential liabilities that require experimental validation before their further development can be assessed.

## 4. Materials and Methods

### 4.1. Plant Samples

Four halophyte plant species (*L. guyonianum*, *S. fruticosa*, *S. vera*, and *H. portulacoides*) were collected on the Kerkennah Islands (34°42′ N, 11°11′ E; Gulf of Gabès, Tunisia) in spring, summer, or winter of 2023. The aerial parts were shade-dried at 40 °C and then ground in a mill until a fine powder was obtained, thus giving rise to nine different samples (TN1-TN9) (see Table 1 for details).

### 4.2. Plant Extracts

The “single-step extraction method for the rapid preparation of polar and semi-polar metabolites from plant samples”, based on a protocol previously described [51], was used. Briefly, a 25 mg aliquot of each sample was placed in microcentrifuge tubes. A fixed volume (1 mL) of extraction solvent M1 (methyl tert-butyl ether:methanol 3:1, *v*/*v*) was added to the homogenized tissues. After adding the extraction solvent, the tubes were vortexed for 1 min and then incubated on an orbital shaker for 45 min at 4 °C, followed by a 15 min sonication step. For phase separation, a volume of 650 μL of solvent M2 (water:methanol 3:1, *v*/*v*) was added to each vial/tube and the samples were vortexed again for 1 min. The samples were then centrifuged at 20,000× *g* for 10 min at 4 °C. After centrifugation, two phases were obtained: a polar phase and a semi-polar phase, separated by a thin interphase. The polar and semi-polar phases were separated by pipetting into independent microcentrifuge tubes and stored at −20 °C to prevent evaporation.

### 4.3. Mass Spectrometry-Based Putative Annotation of Metabolites in Plant Extracts and Acquisition of Reference Compounds

Metabolites in the plant extracts were profiled by ultra-high-resolution mass spectrometry (hybrid Q-TOF with trapped ion mobility spectrometry, TIMS-TOF FLEX, Bruker Daltonics, Bremen, Germany), using both positive and negative ionization modes. Data were acquired in full-scan mode, without MS/MS fragmentation. Candidate compounds were evaluated based on accurate-mass measurements (mass error < 5 ppm) and matching against two natural product libraries: the NIST Research Library (National Institute of Standards and Technology, USA) and the Natural Products Library (Centre of Excellence for Innovative Medicines Research, Andalusia, Spain). The analysis was carried out at the University Institute of Materials Science (ICMUV), Universidad de Valencia, Spain. The available analytical data supporting the putative annotation of the three selected compounds (caffeic acid, demethylzeylasteral, and protocatechuic aldehyde), including retention times, mass-related information, and library-match results, are provided in Supplementary Table S3.

Following the phytochemical analysis, compounds showing a similarity score below 70% in the natural products library were excluded. Primary metabolites were then discarded to prioritize secondary metabolites. No MS/MS fragmentation analysis or confirmation using authentic reference standards was performed. Therefore, the reported metabolite identities should be regarded as tentative MS-based annotations rather than confirmed identifications. In addition, compounds reported in the literature as toxic and/or synthetic were excluded, and only naturally occurring compounds were considered.

After phytochemical analysis, the selected compounds were purchased from MedChemExpress (Sollentuna, Sweden) and Merck (Darmstadt, Germany) for further studies. They were dissolved in DMSO to prepare stock solutions at initial concentrations of 100 mM. Stock solutions were aliquoted and stored at −20 °C until use to avoid repeated freeze–thaw cycles.

### 4.4. Parasites and Experimental Infections

For the efficacy assays, the *L. infantum*-iRFP strain was employed. This parasite line was generated from the *L. infantum* BCN150 isolate (MCAN/ES/96/BCN150) and was previously developed in our laboratory to constitutively express the iRFP. Stable iRFP expression enables the detection and quantification of viable parasites by near-infrared fluorescence imaging, as previously described [52]. Promastigotes were routinely cultured at 26 °C under continuous shaking in Schneider’s insect medium (Sigma-Aldrich, Merck, Darmstadt, Germany) supplemented with 20% fetal bovine serum (FBS), 100 U/mL penicillin, and 100 μg/mL streptomycin [53].

For the generation of axenic amastigotes, eight-week-old female BALB/c mice were infected intraperitoneally with 2.5 × 10^9^ metacyclic promastigotes of the *L. infantum*-iRFP strain. Eight weeks post-infection, animals were humanely euthanized, and the femurs and tibias from both hind limbs were aseptically collected. Bone marrow cells recovered from the medullary cavities were processed to obtain *L. infantum*-iRFP axenic amastigotes according to the protocol previously described [54]. Briefly, cells were incubated at 37 °C in a humidified atmosphere containing 5% CO_2_ in amastigote culture medium composed of 15 mM KCl, 136 mM KH_2_PO_4_, 10 mM K_2_HPO_4_·3H_2_O, 0.5 mM MgSO_4_·7H_2_O, 24 mM NaHCO_3_, 22 mM glucose, 1 mM glutamine, RPMI 1640 vitamin mixture (1×; Sigma-Aldrich, Merck, Darmstadt, Germany), 10 μM folic acid, 100 μM adenosine, RPMI amino acid mixture (1×; Sigma-Aldrich, Merck, Darmstadt, Germany), 5 μg/mL hemin, and antibiotic cocktail, 25 mM MES, and 20% FBS, allowing their differentiation into axenic amastigotes.

### 4.5. Efficacy Assays Against L. infantum-iRFP Axenic Amastigotes

A total of 3 × 10^4^ iRFP-expressing *L. infantum* axenic amastigotes were seeded per well in 40 μL of amastigote culture medium in 384-well microplates. Amphotericin B (10 μM) was used as the positive control, whereas 0.1% (*v*/*v*) DMSO served as the negative control.

To test the bioactivity of plant extracts, polar and semi-polar extracts were added in a final volume of 10 μL, and the volume in each well was adjusted to 80 μL with amastigote culture medium. Additional control wells containing the corresponding solvent mixtures (M1 and M2, see Section 4.2) were also included.

The antileishmanial activity of the purified compounds was initially assessed by single-concentration screening at 20 and 1 μM. Compounds producing a significant reduction in parasite viability (>70%) were subsequently selected to determine their EC_50_ values using a range of increasing concentrations up to 10 μM (demethylzeylasteral) or 100 μM (caffeic acid and protocatechuic aldehyde) dissolved in the amastigote culture medium and adjusting the final volume to 80 μL.

Plates were incubated for 72 h at 37 °C in a humidified atmosphere containing 5% CO_2_. Parasite viability was determined by measuring the fluorescence emitted by the iRFP-expressing parasites using an Odyssey Infrared Imaging System (LI-COR Biosciences, Lincoln, NE, USA). Fluorescence intensity at 700 nm was recorded at 0, 24, 48, and 72 h, with the plates returned to the incubator between measurements. Parasite viability was expressed relative to the fluorescence of the controls, assigning 100% viability to parasites treated with 0.1% (*v*/*v*) DMSO and 0% viability to those exposed to 10 μM amphotericin B. Three independent biological replicates carried out in duplicate were performed.

### 4.6. Cell Cytotoxicity

The cytotoxicity of the plant extracts and chemical compounds was evaluated in fully developed mouse intestinal organoid cultures (day 4 of growth), which were obtained, cultured and adapted to a 384-well format as previously described [55]. Aliquots (8 μL) were dispensed into each well, followed by matrix polymerization and addition of 32 μL of culture medium. Then, they were treated with 10 μL of plant extract fractions or with 10 μL of serial dilutions of the compounds of interest dissolved in organoid culture medium, and the volume in each well was adjusted to 80 μL with organoid culture medium. For compound testing, 1:2 serial dilutions were prepared, ranging from 100 μM to 1.56 μM (demethylzeylasteral and protocatechuic aldehyde) or from 200 μM to 0.05 μM (caffeic acid) to calculate the CC_50_ values.

Cultures were incubated for 72 h at 37 °C in a humidified atmosphere containing 5% CO_2_. Hydrogen peroxide (0.15% *v*/*v*) was used as the positive control, whereas 0.2% (*v*/*v*) DMSO served as the negative control. After the incubation period, cell viability was determined using the alamarBlue assay (Thermo Fisher Scientific, Waltham, MA, USA). A volume equivalent to 10% of the well content was added to each well, and plates were incubated for an additional 4 h at 37 °C and 5% CO_2_. Fluorescence, which is proportional to cell viability, was measured using a Varioskan Lux plate reader (Thermo Fisher Scientific) with excitation and emission wavelengths of 560 and 590 nm, respectively. Results were normalized to the corresponding controls, assigning 100% viability to organoids treated with 0.2% (*v*/*v*) DMSO and 0% viability to those exposed to hydrogen peroxide and expressed as percentage cell viability. Three independent biological replicates carried out in duplicate were performed.

### 4.7. Dose–Response Analysis and SI Determination

Dose–response curves were generated by plotting cell viability (parasites and organoids) as a function of compound concentration. EC_50_ (efficacy) and CC_50_ (cytotoxicity) values were calculated by non-linear regression analysis using SigmaPlot version 10.1. The SI was calculated as the ratio of the CC_50_ to the EC_50_ values.

### 4.8. TryR Colorimetric Inhibition Assay

TryR activity was determined using a colorimetric assay based on the reduction of 5,5′-dithiobis-(2-nitrobenzoic acid) (DTNB; Alfa Aesar, Fisher Scientific, Inc., Waltham, MA, USA), as previously described [56]. Enzyme extracts were prepared from 1 × 10^10^ *L. infantum*-iRFP promastigotes harvested at the late logarithmic growth phase. Briefly, 25 μL of enzyme extract was dispensed into each well, followed by the addition of 1 μL of the test compound at different concentrations. Wells containing 2.5% (*v*/*v*) DMSO served as the negative control, whereas 100 μM thioridazine (MedChemExpress), a well-known potent inhibitor of TryR [57], was used as the positive control. The negative control showed a specific enzymatic activity of 1.83 × 10^−2^ μmol/mg · min. (ΔA/t = ɛ · d · c; ɛ = 14.150 M^−1^ cm^−1^; d = 0.34 cm).

The reaction was initiated by adding 14 μL of reaction mixture containing 200 μM NADPH, 75 µM TS_2_, 75 µM DTNB, and 50 mM Tris-HCl buffer (pH 7.5). Absorbance at 412 nm was recorded every 5 min for 2 h at 27 °C using a Varioskan LUX microplate reader (Thermo Fisher Scientific).

### 4.9. Predictive Drug-likeness and ADMET

Physicochemical properties, drug-likeness, gastrointestinal absorption, blood–brain barrier permeability, CYP450 inhibition, and medicinal chemistry parameters were predicted using SwissADME [58]. Toxicological endpoints, including Ames mutagenicity, hepatotoxicity, and oral rat acute toxicity (LD_50_), were predicted using pkCSM [59].

## 5. Conclusions

The present study highlights *S. vera* as a promising source of antileishmanial metabolites and reports the putative annotation of secondary metabolites in its extracts. Caffeic acid, demethylzeylasteral, and protocatechuic aldehyde, selected based on the phytochemical profiling, exhibited antileishmanial activity when evaluated individually, although their contribution to the activity of the original extracts remains to be established. None of these compounds inhibited TryR-associated activity in the assay performed using parasite protein extracts, although direct inhibition of the purified enzyme cannot be excluded. In silico analyses revealed distinct predicted pharmacokinetic and toxicity profiles, with caffeic acid showing a promising overall profile despite potential liabilities requiring further investigation. These findings support further studies to clarify the molecular mechanisms underlying the antileishmanial activity of these compounds and to assess their therapeutic potential in vivo.

## Figures and Tables

**Table 1:** Samples analyzed in this work.

Sample	Plant Species	Family	Harvest Season
TN1	*Limoniastrum guyonianum* Boiss.	Plumbaginaceae	Summer 2023
TN2	*Limoniastrum guyonianum* Boiss.	Plumbaginaceae	Spring 2023
TN3	*Sarcocornia fruticosa* (L.) A.J.Scott (≡*Salicornia europaea* var. *fruticosa* L.)	Amaranthaceae	Summer 2023
TN4	*Sarcocornia fruticosa* (L.) A.J.Scott (≡*Salicornia europaea* var. *fruticosa* L.)	Amaranthaceae	Spring 2023
TN5	*Suaeda vera* Forssk. ex J.F.Gmel.	Amaranthaceae	Summer 2023
TN6	*Suaeda vera* Forssk. ex J.F.Gmel.	Amaranthaceae	Spring 2023
TN7	*Halimione portulacoides* (L.) Aellen	Amaranthaceae	Summer 2023
TN8	*Halimione portulacoides* (L.) Aellen	Amaranthaceae	Spring 2023
TN9	*Suaeda vera* Forssk. ex J.F.Gmel.	Amaranthaceae	Winter 2023

**Table 2:** Putatively annotated secondary metabolites in the bioactive and non-cytotoxic *S. vera* extracts. Compounds are listed in alphabetical order, indicating the main chemical class and subclass.

Compound	Main Chemical Class	Subclass
2′-Hydroxy-4,4′,6′-trimethoxychalcone	Flavonoid	Chalcone
4′-Hydroxyacetophenone	Phenolic compound	Phenolic acetophenone
4-Hydroxybenzoic acid	Phenolic compound	Hydroxybenzoic acid
4-Methylcinnamic acid	Phenylpropanoid	Cinnamic acid derivative
5,6-Dehydroarachidonic acid	Lipid	Polyunsaturated fatty acid derivative
5-Hydroxy-3,4-dihydro-2(1H)-quinolinone	Nitrogen-containing heterocycle	Dihydroquinolinone
5-Methoxypsoralen (Bergapten)	Coumarin	Linear furanocoumarin
10-Gingerol	Phenolic compound	Gingerol
Asiatic acid	Terpenoid	Pentacyclic triterpenoid
Betulonic acid	Terpenoid	Pentacyclic triterpenoid
Blinin	Terpenoid	Diterpenoid
Caffeic acid	Phenylpropanoid	Hydroxycinnamic acid
Carvacrol	Terpenoid	Phenolic monoterpene
Chikusetsusaponin IVa	Terpenoid	Triterpenoid saponin
Chrysophanol	Phenolic polyketide	Hydroxyanthraquinone
Cinnamic acid	Phenylpropanoid	Cinnamic acid
Columbin	Terpenoid	Furanoditerpene lactone
Daidzin	Flavonoid	Glycosylated isoflavone
Demethylzeylasteral	Terpenoid	Quinone-methide triterpenoid
Engeletin	Flavonoid	Flavanonol glycoside
Glycyrrhetinic acid	Terpenoid	Pentacyclic triterpenoid
Heterophyllin B	Peptide	Cyclooctapeptide
Higenamine	Alkaloid	Benzylisoquinoline alkaloid
Hordenine	Alkaloid	Phenethylamine alkaloid
Hydrocinnamic acid ethyl ester	Phenylpropanoid	Phenylpropanoid acid ester
Hyperin (Quercetin-3-galactoside)	Flavonoid	Flavonol glycoside
Isoimperatorin	Coumarin	Prenylated furanocoumarin
Isoliensinine	Alkaloid	Bisbenzylisoquinoline alkaloid
Isorhamnetin	Flavonoid	Methoxylated flavonol
Isorhamnetin-3-O-neohesperidoside	Flavonoid	Flavonol glycoside
Isorhynchophylline	Alkaloid	Oxindole alkaloid
Isoquercitrin (Quercetin-3-glucoside)	Flavonoid	Flavonol glycoside
Leonurine	Alkaloid	Phenolic guanidine alkaloid
Liriopesides B	Terpenoid	Steroidal saponin
Narcissoside (Isorhamnetin-3-rutinoside)	Flavonoid	Flavonol glycoside
Nodakenin	Coumarin	Glycosylated linear furanocoumarin
Paeonol	Phenolic compound	Phenolic acetophenone
Peiminine	Alkaloid	Steroidal alkaloid
Phytolaccagenin	Terpenoid	Pentacyclic triterpenoid
Picroside II	Terpenoid	Iridoid glycoside
Prim-O-glucosylcimifugin	Chromone	Chromone glycoside
Protocatechuic aldehyde	Phenolic compound	Dihydroxybenzaldehyde
Quercetin	Flavonoid	Flavonol
Quercetin 7-rhamnoside (Vincetoxicoside B)	Flavonoid	Flavonol glycoside
Salidroside	Phenolic glycoside	Tyrosol glycoside
Salsolinol	Alkaloid	Tetrahydroisoquinoline alkaloid
Thymol	Terpenoid	Phenolic monoterpene
trans-3-Coumaric acid	Phenylpropanoid	Hydroxycinnamic acid
Trigonelline	Alkaloid	Pyridine alkaloid (betaine)
Typhaneoside	Flavonoid	Flavonol glycoside
Ursolic acid	Terpenoid	Pentacyclic triterpenoid
Zingerone	Phenolic compound	Methoxyphenolic ketone
Ziyuglycoside	Terpenoid	Triterpenoid saponin

**Table 3:** In vitro efficacy against *L. infantum*-iRFP amastigotes (EC_50_) and safety (CC_50_) in intestinal organoids of caffeic acid, demethylzeylasteral, and protocatechuic aldehyde. SI values are indicated.

Compound	EC_50_ (μM)	CC_50_ (μM)	SI (CC_50_/EC_50_)
Caffeic acid	1.09 ± 0.15	>100	>91.74
Demethylzeylasteral	0.20 ± 0.01	4.78 ± 0.68	23.9
Protocatechuic aldehyde	14.56 ± 1.02	>100	>6.86

**Table 4:** Predictive drug-likeness and ADMET of caffeic acid, demethylzeylasteral, and protocatechuic aldehyde.

Parameter	Caffeic Acid	Demethylzeylasteral	Protocatechuic Aldehyde
**Drug-likeness rules**			
Chemical formula	C_9_H_8_O_4_	C_29_H_36_O_6_	C_7_H_6_O_3_
Molecular weight (Da)	180.16	480.59	138.12
cLogP	0.92	5.16	0.80
Hydrogen bond acceptors	4	6	3
Hydrogen bond donors	3	3	2
Rotatable bonds	2	2	1
Lipinski rule	0 violations	0 violations	0 violations
**Absorption**			
Human Intestinal absorption	High	Low	High
P-glycoprotein substrate	No	Yes	No
Blood–brain barrier	Non-permeant	Non-permeant	Permeant
**Metabolism**			
CYP450 1A2 inhibitor	Yes	No	No
CYP450 2C19 inhibitor	No	No	No
CYP450 2C9 inhibitor	No	No	No
CYP450 2D6 inhibitor	No	No	No
CYP450 3A4 inhibitor	No	No	Yes
**Toxicity**			
Ames test	No	No	Yes
Hepatotoxicity	No	No	No
Oral rat LD_50_	1.996 mol/kg	2.963 mol/kg	1.852 mol/kg
**PAINS alerts**	1: catechol_A	1: catechol A	1: catechol A

## Data Availability

Data supporting reported results can be found in the Zenodo repository at https://doi.org/10.5281/zenodo.23269656.

## Supplementary Materials

The following supporting information can be downloaded at https://www.mdpi.com/article/10.3390/molecules31213967/s1, Table S1: Putatively annotated secondary metabolites in the polar extracts of *S. vera* collected during the spring (TN6), summer (TN5), and winter (TN9) seasons; Table S2: Putatively annotated secondary metabolites in the semi-polar extracts of *S. vera* collected during the spring (TN6), summer (TN5), and winter (TN9) seasons; Table S3: Analytical data supporting the putative annotation of selected compounds in *S. vera* extracts; Figure S1: Time-dependent in vitro inhibition of TryR by different concentrations (25 μM to 100 μM) of caffeic acid (left panel), demethylzeylasteral (middle panel), and protocatechuic aldehyde (right panel).

## Abbreviations

The following abbreviations are used in this manuscript:

3D	three-dimensional
CC_50_	half maximal cytotoxic concentration
DTNB	5,5′-dithiobis-(2-nitrobenzoic acid)
EC_50_	half maximal effective concentration
FBS	fetal bovine serum
iRFP	infrared fluorescent protein
ROS	reactive oxygen species
SI	selectivity index
TryR	trypanothione reductase
TS_2_	oxidized trypanothione
