# Supplementary material for: Phytochemical Profiling of the Halophyte Suaeda vera and Antileishmanial Evaluation of Its Extracts and Selected Metabolites, with In Silico ADMET Analysis
Source: Molecules. 2026 Nov 3;31(21):3967. doi: 10.3390/molecules31213967 (PMC13764740; doi:10.3390/molecules31213967)
Supplement: Supplementary file 1 [file molecules-31-03967-s001.zip › Supplemntary Figure S1.pdf]

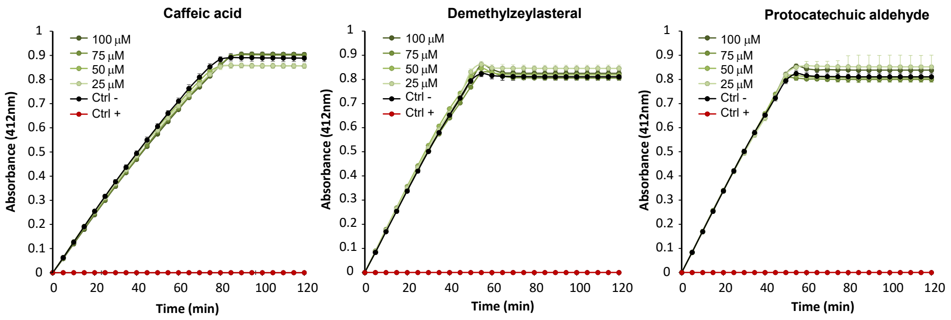

Figure S1. Time-dependent in vitro inhibition of TryR by different concentrations (25 μM to 100 μM) of caffeic acid (left panel), demethylzeylasteral (middle panel) and protocatechuic aldehyde (right panel). Thioridazine (0.1 mM) was used as a positive control (C+), whereas 2.5% (v/v) DMSO was used as negative control (C-). Results show the mean values ± SD of two independent experiments with three technical replicates each.
