# Supplementary material for: Phytochemical Profiling of the Halophyte Suaeda vera and Antileishmanial Evaluation of Its Extracts and Selected Metabolites, with In Silico ADMET Analysis
Source: Molecules. 2026 Nov 3;31(21):3967. doi: 10.3390/molecules31213967 (PMC13764740; doi:10.3390/molecules31213967)
Supplement: Supplementary file 1 [file molecules-31-03967-s001.zip › Supplemntary Table S1.pdf]

Table S1. Putatively annotated secondary metabolites in the polar extracts of *S. vera* collected during the spring (TN6), summer (TN5), and winter (TN9) seasons.

Compound number	Spring (TN6)	Summer (TN5)	Winter (TN9)
1	—	—	10-Gingerol
2	—	4'-Hydroxyacetophenone	—
3	—	—	5-Methoxypsoralen (Bergapten)
4	4-Hydroxybenzoic acid	—	—
5	—	—	Asiatic acid
6	—	—	Betulonic acid
7	—	—	Caffeic acid
8	Cinnamic acid	Cinnamic acid	—
9	—	—	Glycyrrhetinic acid
10	—	—	Ursolic acid
11	Protocatechuic aldehyde	Protocatechuic aldehyde	Protocatechuic aldehyde
12	—	—	Blinin
13	Carvacrol	Carvacrol	—
14	—	—	Chikusetsusaponin IVa
15	Daidzin	—	—
16	Engeletin	—	—
17	—	—	Hydrocinnamic acid ethyl ester
18	—	—	Heterophyllin B
19	—	—	Higenamine
20	—	—	Hyperin (Quercetin-3-galactoside)
21	Hordenine	Hordenine	—
22	Isoquercitrin (Quercetin-3-glucoside)	—	—
23	Isorhamnetin	Isorhamnetin	Isorhamnetin
24	—	—	Isorhamnetin-3-O-neohesperidoside
25	—	—	Isorhynchophylline

26	—	—	Liriopesides B
27	Narcissoside (Isorhamnetin-3-rutinoside)	Narcissoside (Isorhamnetin-3-rutinoside)	Narcissoside (Isorhamnetin-3-rutinoside)
28	—	Paeonol	—
29	—	—	Peiminine
30	Salidroside	—	—
31	—	—	Salsolinol
32	—	—	Trigonelline
33	—	—	Zingerone
