# Supplementary material for: Phytochemical Profiling of the Halophyte Suaeda vera and Antileishmanial Evaluation of Its Extracts and Selected Metabolites, with In Silico ADMET Analysis
Source: Molecules. 2026 Nov 3;31(21):3967. doi: 10.3390/molecules31213967 (PMC13764740; doi:10.3390/molecules31213967)
Supplement: Supplementary file 1 [file molecules-31-03967-s001.zip › Supplemntary Table S2.pdf]

Table S2. Putatively annotated secondary metabolites in the semi-polar extracts of *S. vera* collected during the spring (TN6), summer (TN5), and winter (TN9) seasons.

Compound number	Spring (TN6)	Summer (TN5)	Winter (TN9)
1	—	—	10-Gingerol
2	—	—	2'-Hydroxy-4,4',6'-trimethoxychalcone
3	—	—	5-Hydroxy-3,4-dihydro-2(1H)-quinolinone
4	—	—	4-Methylcinnamic acid
5	—	—	5,6-Dehydroarachidonic acid
6	—	—	Betulonic acid
7	Cinnamic acid	Cinnamic acid	—
8	—	—	trans-3-Coumaric acid
9	—	—	Ursolic acid
10	Protocatechuic aldehyde	Protocatechuic aldehyde	Protocatechuic aldehyde
11	—	—	Blinin
12	—	Carvacrol	—
13	Columbin	—	—
14	Chrysophanol	Chrysophanol	—
15	Demethylzeylasteral	—	—
16	—	—	Hydrocinnamic acid ethyl ester
17	—	—	Phytolaccagenin
18	—	—	Hyperin (Quercetin-3-galactoside)
19	Isoimperatorin	—	—
20	Isoliensinine	—	—
21	Isoquercitrin (Quercetin-3-glucoside)	Isoquercitrin (Quercetin-3-glucoside)	—
22	—	Isorhamnetin	Isorhamnetin
23	—	—	Isorhamnetin-3-O-neohesperidoside

24	Leonurine	Leonurine	—
25	—	Narcissoside	—
26	Nodakenin	Nodakenin	—
27	—	—	Peiminine
28	—	Picroside II	—
29	—	Prim-O-glucosylcimifugin	—
30	Quercetin	—	Quercetin
31	Quercetin 7-rhamnoside; (Vincetoxicoside B)	—	—
32	—	Salidroside	—
33	—	—	Salsolinol
34	—	—	Typhaneoside
35	—	—	Thymol
36	—	—	Zingerone
37	—	—	Ziyuglycoside
