# Supplementary material for: Phytochemical Profiling of the Halophyte Suaeda vera and Antileishmanial Evaluation of Its Extracts and Selected Metabolites, with In Silico ADMET Analysis
Source: Molecules. 2026 Nov 3;31(21):3967. doi: 10.3390/molecules31213967 (PMC13764740; doi:10.3390/molecules31213967)
Supplement: Supplementary file 1 [file molecules-31-03967-s001.zip › Supplemntary Table S3.pdf]

Table S3. Analytical data supporting the putative annotation of selected compounds in *S. vera* extracts.

Library hit	Expected Retention time	Retention time	Adduct / Charge	Precursor Mass	Library Score	Plant sample
Protocatechuic aldehyde	1.81	1.78	[M]-	137.025	98.6	TN9 NO POLAR NEG
	0.79	0.8	[M-H]-	137.026	83.2	TN9 POLAR NEG
	1.81	1.84	[M]-	137.025	98.2	TN9 POLAR NEG
	1.44	1.45	[M]-	137.025	79.6	TN6 NO POLAR NEG
	2.37	2.6	[M-H]-	137.062	73.4	TN6 POLAR NEG
	2.57	2.58	[M]-	137.026	83.9	TN6 POLAR NEG
	1.44	1.45	[M]-	137.025	91.7	TN5 NO POLAR NEG
	2.57	2.56	[M]-	137.026	74.6	TN5 POLAR NEG
Caffeic acid	2.26	2.43	[M-H]-	179.036	87.9	TN9 POLAR NEG
Demethylzeylasteral	1.64	1.64	[M+Na]+	481.26	77.2	TN6 NO POLAR POS

Compound identities are putative annotations based on full-scan mass spectrometry and library matching. MS/MS fragmentation and confirmation using authentic analytical standards were not performed.
